# Vorsorgen für das Lebensende bei alleinlebenden hochaltrigen Menschen: Qualitative Studienergebnisse zu Herausforderungen und Grenzen von Advance Care Planning

**DOI:** 10.1007/s00391-025-02414-8

**Published:** 2025-03-07

**Authors:** Sabine Pleschberger, Paulina Wosko

**Affiliations:** 1 Zentrum für Public Health, Medizinische Universität Wien, Kinderspitalgasse 15/1, 1090 Wien, Österreich; 2 Gesundheit Österreich GmbH, Stubenring 6, 1010 Wien, Österreich

**Keywords:** Einpersonenhaushalte, Hochaltrigkeit, Advance Care Planning, End-of-life care, Qualitative Sekundäranalyse, Ältere Menschen, Advance care planning, End-of-life care, Aged 80 and over, Single-household, Qualitative secondary analysis

## Abstract

**Hintergrund:**

Für das Lebensende vorzusorgen (Advance Care Planning), ist für alleinlebende hochaltrige Menschen besonders wichtig, v. a. wenn sie auch zu Hause sterben möchten. Zwischen Relevanz und aktueller Nutzung von Maßnahmen der Vorsorge besteht aber eine große Diskrepanz, deren Ursachen bei dieser Zielgruppe noch wenig erforscht sind.

**Ziel der Arbeit:**

Erfassen der Perspektiven alleinlebender hochaltriger Menschen auf Maßnahmen der Vorsorge rund um das Lebensende.

**Material und Methoden:**

(Sekundär‑)Analyse von 26 qualitativen Interviews mit alleinlebenden Menschen (Ø 84,3 Jahre) in zwei österreichischen Regionen, basierend auf Daten der Österreichischen Interdisziplinären Hochaltrigenstudie (*n* = 17) und selektiver fokussierter Nacherhebung (*n* = 9). Eine induktive Analyse der wörtlich transkribierten Interviews erfolgte durch Anwendung des thematischen Kodierens.

**Ergebnisse:**

Für Vorsorgeregelungen ist ein klares Anliegen entscheidend, etwa der Verzicht auf lebensverlängernde Maßnahmen. Solche Wünsche finden meist in Patientenverfügungen ihren Ausdruck. Weitere häufige Anliegen sind der Wunsch, bis zuletzt in der häuslichen Umgebung bleiben zu können, sowie Regelungen zu Bestattung und Nachlass. Zahlreiche Hürden für formale Regelungen wurden identifiziert.

**Diskussion:**

Die Heterogenität alleinlebender hochaltriger Menschen hinsichtlich Bereitschaft und Form der Vorsorge für den Verbleib in der häuslichen Umgebung am Lebensende zeigt die Grenzen standardisierter Verfahren auf. Modelle für vorausschauende Kommunikation mit hochaltrigen Menschen im häuslichen Umfeld, die über gesundheitliche Vorausplanung hinausgehen, sind ein ernstzunehmendes Desiderat in Forschung und Praxis.

## Abstract (en)

**Background:**

Despite there is evidence for the need for advance care planning for older people living alone (OPLA), especially if they want to stay at home until the end, utilization of measures for ACP remains rather scarce. The causes for this discrepancy have not yet been researched in this target group.

**Objectives:**

Aim of this study was to get insight into the perspectives of OPLA in the community on planning ahead for the end of life.

**Materials and methods:**

(Secondary) Analysis of 26 qualitative interviews. 17 were from the Austrian Survey on the very old (ÖIHS), and 9 additional interviews were conducted with a specific focus on advance care planning to enrich the data base. Verbatim transcripts were analysed by applying coding procedures.

**Results:**

Not all people had specific concerns or a desire to deal with the end of life. In contrast to others, concerns about avoiding (unnecessary) life-prolonging measures resulted in a formal regulation, usually a living will. Staying at home or issues regarding burial and inheritance were often equally important concerns for OPLA. Numerous obstacles to formal arrangements were identified.

**Conclusion:**

The heterogeneity of OPLA with regard to their willingness and form of planning ahead for staying at home shows the limits of standardized procedures. Comprehensive approaches to advance care planning that go beyond health issues are needed to meet the concerns of OPLA in the community.

Für alleinlebende hochaltrige Menschen ist Vorsorge für das Lebensende besonders wichtig, v. a. wenn sie auch zu Hause sterben möchten. Zwischen Relevanz und aktueller Nutzung von Maßnahmen der Vorsorge besteht aber eine große Diskrepanz. Mit der Erforschung von Gründen aus der Sichtweise der betroffenen Menschen soll eine wissenschaftliche Grundlage für die Entwicklung nutzerorientierter Konzepte der Vorausplanung geschaffen werden.

## Hintergrund und Fragestellung

Im höheren Alter lebt ein zunehmender Anteil von Menschen allein in einem Haushalt: In Österreich lebten im Jahr 2023 mehr als die Hälfte (57,8 %) der Frauen, die das 80. Lebensjahr überschritten hatten, allein. Der Anteil der alleinlebenden Männer liegt in derselben Alterskohorte bei 26,5 % [17]. Trotz der Herausforderungen, die diese Lebensform im hohen Alter mit sich bringt, ist es für alleinlebende hochaltrige Menschen ein Anliegen, bis zum Lebensende in ihrer vertrauten Umgebung zu bleiben [5]. Dies erfordert ein tragfähiges Unterstützungssystem formeller und informeller Hilfen, häufig auch außerfamiliärer Natur [6, 10, 11]. In Notfallsituationen bzw. in der letzten Lebensphase können aufgrund des Fehlens anderer vertrauter Personen im Haushalt Entscheidungen über indizierte Maßnahmen erschwert sein bzw. Interventionen erfolgen, die den Wünschen der Betroffen entgegenstehen (z. B. eine Krankenhauseinweisung). Daraus ergibt sich eine besonders hohe Relevanz für Vorsorgeplanung bei dieser Zielgruppe.

Im deutschsprachigen Raum gibt es diverse Optionen zur individuellen Vorsorge, etwa Patientenverfügungen und Vertretungsregelungen. Zudem wird das Modell der gesundheitlichen Versorgungsplanung („Advance Care Planning“, ACP) insbesondere in der stationären Langzeitpflege erprobt [1, 8, 12, 18]. Der Prozess der Auseinandersetzung erfolgt hierbei in Form von Gesprächen zur Exploration der Werthaltungen mit möglichst verbindlicher Dokumentation der Ergebnisse [3, 11]. Allen Ansätzen gemeinsam ist ein Fokus auf medizinische Interventionen am Lebensende. In den vergangenen Jahren ist die Inanspruchnahme von Vorsorgemaßnahmen zwar angestiegen, sie bleibt jedoch besonders bei älteren Menschen ohne schwerwiegende Erkrankung relativ gering [16].

Um alleinlebenden hochaltrigen Menschen den Verbleib zu Hause zu ermöglichen, ist es wichtig, Diskrepanzen zwischen Bedarf, Relevanz und Nutzung vorhandener Vorsorgemaßnahmen besser zu verstehen. Ziel der Studie war es daher, ein differenziertes Verständnis der Perspektiven der Betroffenen auf Vorsorgemaßnahmen zu gewinnen.

## Design & Methode

Das qualitative Design der Studie basiert auf einer Sekundäranalyse [19] mit einer ergänzenden Nacherhebung. Als Primärstudie diente die zweite Welle der Österreichischen Interdisziplinären Hochaltrigenstudie (ÖIHS) 2015–2018, mit der seit 2013 regelmäßig umfangreiche Daten zu Gesundheit, Mobilität, sozialen Aspekten und der Lebenssituation hochaltriger Menschen (≥ 80 Jahre) in Österreich erhoben werden [13]. Die Panel-Erhebung wird durch qualitative Fokusstudien zu wechselnden Schwerpunktthemen ergänzt, darunter offene Fragen zu Zukunftsperspektiven und zum Lebensende. Von den 40 transkribierten und anonymisierten Interviews der ÖIHS wurden jene extrahiert und zur Analyse übermittelt, die mit alleinlebenden Menschen in der häuslichen Umgebung geführt wurden (*n* = 17). Aus ethischen Gründen erhielten die Autorinnen weder Kontaktdaten noch personenbezogene Informationen. Einige Befragte lebten in einer Seniorenresidenz mit eigenem Appartement oder gaben erst im Interview die räumliche Distanz zu Angehörigen an. Diese Fälle wurden nach sorgfältiger Prüfung in die Stichprobe aufgenommen und teils kontrastierend analysiert. Dadurch wurde die Perspektive auf den Umgang mit Angehörigen und nichtfamiliären Helfer*innen erweitert. In keinem Fall wohnten Angehörige im selben Haushalt. Die Interviews dieser Studie wurden mit dem Fokus auf das Lebensende und mögliche Vorsorgemaßnahmen entlang eines Kategorienschemas thematisch (re)kodiert [4]. Die induktive Analysemethode des thematischen Kodierens wurde gewählt, um durch konstante Vergleiche innerhalb der Stichprobe Gemeinsamkeiten, Unterschiede sowie Muster zu identifizieren [4]. Die Daten der Primärstudie boten nicht in allen Themenfeldern ausreichende Differenzierung. Zudem wurde gemäß der qualitativen Sampling-Strategie eine größtmögliche Vielfalt hinsichtlich des Merkmals „alleinlebend“ (Familiensituation) angestrebt. Deshalb erfolgte eine selektive Nacherhebung (*n* = 9) im großstädtischen Umfeld sowie in einer ländlichen Region. Über „Gatekeeper“ wie Seniorenvereine oder Kirchengemeinschaften sowie durch das Schneeballverfahren wurden gezielt Interviewpartner*innen gesucht, die keine Familie im Nahbereich hatten (mindestens 25 km Entfernung). Kontaktdaten wurden erst nach Zustimmung der potenziellen Teilnehmer*innen an die Forscherinnen weitergegeben. Ein schriftlicher Informed Consent wurde vor jedem Interview nach ausführlicher Information über die Zusicherung von Anonymität, Vertraulichkeit und Einhaltung der Datenschutzbestimmungen eingeholt.

Der Leitfaden für die Nacherhebung wurde sowohl literatur- als auch ergebnisbasiert (Analyse der Primärstudie) ergänzt (Infobox). Die Frage nach möglichen „Regelungen“ wurde bewusst offen formuliert, um bei Nachfragen einzelne Maßnahmen differenziert erheben zu können. Die Interviews dauerten im Schnitt etwa 50 min; sie wurden digital aufgezeichnet und wörtlich transkribiert. Für die Analyse wurde das oben genannte Kategorienschema weitergeführt und induktiv erweitert. Die Zuordnungen und Gruppierungen wurden im Autorinnenteam gemeinsam diskutiert und so validiert.

### Infobox Exemplarische Fragen im Leitfaden der Nacherhebung

- Wenn Sie nach vorne schauen in die Zukunft, für sich selbst und Ihre Lebenssituation: Was beschäftigt Sie da?
- Es gibt ja Möglichkeiten, vorab bestimmte Dinge zu regeln für den Fall bzw. die Zeit, wo man das nicht mehr selbst bestimmen kann. Haben Sie davon schon gehört?
- Welche Dinge wären Ihnen wichtig, vorab zu regeln, für den Fall, dass Sie selbst einmal nicht mehr bestimmen könnten?
- Mit wem sprechen Sie darüber?

## Ergebnisse

Die Datengrundlage für diese Studie umfasst somit 26 Einzelinterviews (17 ÖIHS + 9 aus selektiver Nacherhebung) mit alleinlebenden hochaltrigen Menschen aus Österreich in Einpersonenhaushalten bzw. Seniorenresidenzen mit Einzelappartements, für deren Nutzung kein bestimmter Grad von Pflegebedürftigkeit erforderlich war (Tab. 1).

**Tab. 1:** Stichprobenbeschreibung (*n* = 26)

	Anzahl (*n*)
*Alter in Jahren*	
Range: 80–96 (Ø 84,3)	
*Geschlecht*	
Weiblich	19
Männlich	7
*Wohnform*	
Privathaushalt	21
Seniorenresidenz	5
*Bildungshintergrund*	
Akademischer Abschluss	8
Matura	7
Fachschule ohne Matura	3
Lehre	5
Pflichtschule	3
*Familienstand*	
Verwitwet	12
Geschieden	8
Nie verheiratet	5
k. A.	1
*Kinder*	
Keine Kinder	8
Vorhanden > 25 km entfernt	12
Vorhanden ≤ 25 km entfernt	5
k. A.	1

Die befragten hochaltrigen Menschen standen der Idee, für das Lebensende vorzusorgen, grundsätzlich positiv gegenüber, und einige hatten entsprechende Maßnahmen getroffen. Eine klare Differenzierung, wofür genau, und mit welcher Maßnahme vorgesorgt wurde, erfolgte jedoch nur in Ausnahmefällen.

Gespräche über Vorsorge fanden v. a. im Freundes- und Bekanntenkreis statt, oft ausgelöst durch Todesfälle und ähnliche Ereignisse im Umfeld. Mitunter forderten auch Angehörige eine Regelung ein. Befragte mit familiären Kontakten berichteten von ambivalenten Erfahrungen: Oft war es schwierig, Sorgen und Ängste offen zu besprechen, oder es bestand die Sorge, Angehörige mit (zu) hohen Erwartungen zu belasten, was zu Zurückhaltung führte. Hausärzt*innen wurden als potenzielle Ansprechpersonen für Anliegen rund um das Lebensende genannt, ihre Haltung wurde jedoch unterschiedlich wahrgenommen. Besonders wenn keine fortschreitende Erkrankung vorlag, war es keineswegs selbstverständlich, sich an sie zu wenden.

„Wirklich, so wie ich mit Ihnen sprechen kann, sind sehr wenige. Da ist nur eine da, die hat studiert, aber die spricht überhaupt mit niemand, ich weiß nicht, die geht immer alleine, will sie mit niemandem reden?“ (Int. 17)

### Vorsorgeaktivitäten und Anliegen

Von den 26 interviewten Personen hatten sechs eine formalisierte Vorsorge für das Lebensende getroffen: Fünf errichteten eine Patientenverfügung, eine Person eine Vorsorgevollmacht. Solche Regelungen betrafen hauptsächlich das Anliegen der Vermeidung lebensverlängernder Maßnahmen (a). Bei Personen mit familiären Beziehungen erfolgte dies meist auf Initiative der Angehörigen. Weitere Themen die in Gesprächen zur Vorsorge behandelt wurden, umfassten das zentrale Anliegen bis zum Lebensende zu Hause bleiben zu können (b), Anliegen bezüglich Regelungen zur Bestattung (c) und Anliegen im Umgang mit dem Nachlass (d). Diese Anliegen ließen sich jedoch nicht direkt mit gängigen Vorsorgemaßnahmen verknüpfen (s. unten).

### Gründe für fehlende Vorsorge trotz Anliegen und Auseinandersetzung

Einige Personen äußerten Anliegen zum Lebensende, hatten jedoch keine vorsorgenden Maßnahmen ergriffen. Identifizierte Barrieren bezogen sich sowohl auf formalisierte Angebote als auch auf grundsätzliche Bedenken gegenüber einer „Vorsorge für das Lebensende“.

#### Fehlende Information und administrative Hürden

Für das Errichten einer formellen Vorausverfügung müssen die Möglichkeiten bekannt sein. In den Interviews wurden Vorsorgemaßnahmen häufig nur mit Patientenverfügungen assoziiert. Einige Befragte hatten sich aktiv über Vorsorgemaßnahmen informiert, z. B. über das Internet oder Hospizvereine. Das folgende Zitat ist exemplarisch für diese Gruppe:

„Ich habe darüber gelesen irgendwo in der Zeitung – habe sofort dort angerufen, die haben mir das geschickt, und dann liegt’s jetzt in der Lade.“ (Int. 4)

Die Interviewpartnerin gab an, nicht mehr daran zu denken, weshalb sie das Thema fallen gelassen habe. In anderen Interviews fanden sich jedoch Hinweise darauf, warum zugesandte Materialien nicht in eine Verfügung mündeten: Die Informationsfülle und die administrativen Schritte schienen für die hochaltrigen Menschen oft eine zu große Hürde zu sein:

„Im Internet habe ich das einmal gesehen, und das ist so ein Packerl. Also das ist da sehr rigoros. Kann man nicht leichtfertig machen.“ (Int. 9).

#### Eignung der Instrumente

Ein konkretes und wiederkehrendes Anliegen in den Interviews war der Wunsch, einen Heimeintritt bei zunehmender Hilfe- und Pflegebedürftigkeit zu vermeiden, und bis zuletzt zu Hause bleiben zu können. Dieser Wunsch wurde teilweise sehr eindringlich geäußert, wie das folgende Beispiel verdeutlicht:

„Und mein Vater ist zu Hause gestorben, meine Mutter ist zu Hause gestorben, mein Mann ist zu Hause gestorben, und ich *muss* auch hier sterben!“ (Int. 3)

In keinem der Interviews konnte dieser Wunsch mit einer formalen Vorsorgemaßnahme in Verbindung gebracht werden. Dieses Phänomen haben wir als „fehlende Eignung der für die Betroffenen verfügbaren Instrumente“, wie z. B. der Patientenverfügung, kategorisiert.

#### Finanzielle Aspekte

Die mit der Errichtung einer Patientenverfügung verbundenen Kosten wurden als weitere Barriere identifiziert. Allerdings wurde die eigene ökonomische Situation in keinem Interview offen besprochen. Stattdessen wurden finanzielle Aspekte oft mit anderen Argumenten verknüpft, etwa der Frage nach der Sinnhaftigkeit formaler Vorsorge, wie das folgende Zitat zeigt:

„Nein, das mach ich nicht, weil sie erstens nicht gelesen […] und zweitens nicht eingehalten wird. Ach, da können Sie machen, was Sie wollen. Es wird dann immer auf Erste Hilfe geschaltet, verstehen Sie mich. Das wird vorher […] nicht durchgelesen […]. Obendrein ist das sehr schwierig. Man muss das über ein Notariat machen und muss immer 150 € hinlegen, […] ist viel zu teuer. Also das mach ich auf keinen Fall. Ich hab genau mich erkundigt.“ (Int. 6)

Ob die Kosten generell als zu hoch empfunden wurden, als unverhältnismäßig angesichts bestehender Zweifel an der Wirksamkeit, oder ob dies mit der eigenen finanziellen Lage zusammenhing, bleibt an dieser Stelle offen.

#### Religiöse/spirituelle Aspekte

Des Weiteren können religiöse Gründe gegen die Errichtung einer Verfügung sprechen; zum Beispiel wurde es von manchen Befragten als anmaßend angesehen, den Lauf der Dinge beeinflussen zu wollen. Beispielsweise äußerte eine Interviewpartnerin an mehreren Stellen zwar klar den Wunsch, sterben zu dürfen, wenn es soweit wäre, also keine lebensverlängernden Maßnahmen zu wollen, aber einer Patientenverfügung stand sie skeptisch gegenüber. Auf Nachfrage begründete sie dies wie folgt:

„Man denkt, Gott lenkt. (…) Keine lebensverlängernden Maßnahmen.“ (Int. 18)

Gleichzeitig gab es auch Personen in der Stichprobe, die sich als religiös bezeichneten und dennoch eine Patientenverfügung verfasst hatten.

### Gründe für eine fehlende Auseinandersetzung mit Vorsorge

Von den zwölf Personen, die sich noch nicht näher mit den Themen auseinandergesetzt hatten, äußerte die Mehrheit keine drängenden Anliegen. Vielmehr schienen sie auf den natürlichen Lauf der Dinge oder nahestehenden Personen zu vertrauen, ohne mit diesen jedoch konkret darüber gesprochen zu haben. Bei jenen, die auch auf Nachfrage im Interview nicht näher Stellung zum Thema genommen haben, konnten drei Gründe identifiziert werden:

#### Anderer Fokus

Die Sinnhaftigkeit, sich mit Fragen des Lebensendes zu beschäftigen, wurde zwar nicht abgesprochen, nur schienen andere Themen zum Zeitpunkt der Befragung wichtiger.

#### Scheu und Abwehr

Die Auseinandersetzung wurde abgelehnt, weil dadurch Dinge heraufbeschworen werden könnten. Eine Konfrontation mit Sterben und Tod wurde als bedrohlich empfunden:

„Nein, hab’ ich eigentlich nicht nachgedacht. Ich hab’ auch noch kein Testament gemacht. Bin ich ein wenig abergläubisch. Wenn ich das Testament mach’, zum Schluss sterb’ ich gleich. Das will ich nicht. Ich möcht’ ja noch ein bisschen leben.“ (Int. 21)

#### Wozu Gedanken machen?

Auf den ersten Blick signalisiert diese Einstellung Gleichgültigkeit gegenüber Vorsorgefragen. Auf die Thematik einer möglichen (zunehmenden) Hilfe- und Pflegebedürftigkeit hatten sich diese Interviewpersonen jedoch sehr wohl eingelassen. Daher könnte diese Haltung auch als Verdrängung oder als Ausdruck der Schwierigkeit, sich mit dem eigenen Tod auseinanderzusetzen, interpretiert werden:

„I: Und haben Sie sich schon einmal mit dem Thema Sterben und Tod auch irgendwie beschäftigt, was Sie sich wünschen für Sie selbst, wenn es dann einmal zu Ende geht?

IP: Eigentlich nicht, nein. Ich denke mir immer wieder, der Wunsch, weil Sie das jetzt anschneiden, mein Wunsch wäre, dass ich so einschlafe, wie meine Frau. Das ist das Einzige. (…) Denke ich mir, warum sollte ich mir darüber Gedanken machen? Gedanken mache ich mir, wie geht es dir? Wirst du die Gesundheit erhalten? Oder eben: Bist du jahrelang ans Bett gebunden?“ (Int. 26)

## Diskussion

In dieser Studie wurden erstmals hochaltrige Menschen in Einpersonenhaushalten unabhängig von einem Krankheitsbild zu ihren Perspektiven auf Vorsorge für das Lebensende befragt. Die Ergebnisse unterstreichen, dass die Befragten solchen Gesprächen überwiegend positiv gegenüberstehen, jedoch oft keine geeigneten Ansprechpersonen dafür finden. Alleinlebende ältere Menschen äußern ein starkes Verantwortungsgefühl und das Bedürfnis andere zu entlasten, weshalb sie auf verschiedenen Ebenen vorsorgen möchten [9, 15].

Während es für stationäre Pflege- und Betreuungseinrichtungen erprobte Modelle und im Zusammenhang mit dem Rechtsanspruch auf ACP in Deutschland (§ 132g Abs. 3, SGB V) zunehmend auch in finanzieller Hinsicht etablierte Praxis gibt, fehlt diese im häuslichen Bereich noch weitgehend. Wenig überraschend werden daher die Themen Sterben und Tod im Freundes- und Bekanntenkreis oft anlassbezogen verhandelt, wie die Ergebnisse dieser Studie gezeigt haben. Informelle außerfamiliäre Helfer*innen sind für alleinlebende Menschen häufig sehr wichtig, und sie übernehmen auch anwaltschaftliche Aufgaben und Rollen rund um das Lebensende [9]. Damit die Anliegen der Betroffenen auch nachhaltig und rechtlich verbindlich durchgesetzt werden können, bedarf es allerdings formaler Regelungen, insbesondere wenn nur außerfamiliäre Bezugspersonen vorhanden sind [9]. Dass ältere Menschen ihre Anliegen oft nicht „absichern“ und sich somit in rechtlicher Hinsicht in einer „Scheinsicherheit“ wiegen, ist als Phänomen auch im Kontext von Pflegeheimen bekannt [21]. In einer australischen Studie konnte gezeigt werden, dass Gespräche mit Ärztinnen und Ärzten die Wahrscheinlichkeit für ein vollständiges ACP um das Dreifache erhöhten [15].

Die Daten dieser Studie deuten darauf hin, dass der soziale Druck zur Vorsorge gestiegen ist. Bei Befragten, die eine Patientenverfügung errichtet hatten, geschah dies häufig auf Drängen von Angehörigen oder aufgrund von Nachfragen in Krankenhäusern, nicht zuletzt deshalb, weil formale Regelungen auch von den Angehörigen der Gesundheitsberufe als entlastend erlebt werden [7]. Nicht alle Menschen möchten sich jedoch aktiv mit dem Lebensende auseinandersetzen. Diese Ergebnisse stimmen mit anderen Arbeiten überein [2, 16]. Für andere wiederum sind die administrativen Hürden zu hoch, insbesondere bei der Errichtung einer Vorsorgevollmacht (gesetzliche Vertretungsregelung). Zudem ist der Zeitpunkt oder Anlass für Gespräche über das Lebensende schwer zu bestimmen, wenn kein progredientes Krankheitsgeschehen, sondern Gebrechlichkeit im hohen Alter im Vordergrund steht [16].

Von einem Verständnis über Wesen und Inhalte der gesundheitlichen Vorausplanung kann bei den alleinlebenden hochaltrigen Menschen nicht ausgegangen werden, ähnlich wie in einer britischen Studie gezeigt wurde [2]. Allenfalls die Sorge vor „unnötigen“ Lebensverlängerungen muss hier erwähnt werden. In unserem Sample stand die Errichtung von Patientenverfügungen ausschließlich in diesem Zusammenhang. Ähnlich wie in einer Studie zu ACP bei hochbetagten Personen mit fortgeschrittener Herzinsuffizienz [23] sind auch bei den Befragten unserer Studie Zweifel angebracht, ob die Inhalte der Patientenverfügung wirklich verstanden wurden. Dies unterstreicht die Notwendigkeit, die Gesundheitskompetenz zu Themen des Lebensendes zu fördern, insbesondere bei älteren und hochaltrigen Menschen [22].

Ein zentrales Ergebnis dieser Studie ist schließlich, dass die wesentlichen Anliegen von alleinlebenden älteren Menschen in den bekannten Instrumenten der Vorsorge, wo der Fokus in erster Linie auf therapeutischen Interventionen am Lebensende liegt, nicht aufgenommen werden können: Die Sorge, ob und wie sie bei fortschreitender Hilfe- und Pflegebedürftigkeit zu Hause bleiben können, beschäftigte die meisten Befragten, ebenso wie Aspekte der Bestattung sowie der Nachlassregelung. Dies verdeutlicht den Bedarf an Angeboten der Vorsorge, in denen sowohl existenziellen als auch gesundheitsbezogenen Anliegen Raum gegeben wird [14].

## Limitationen

Wie bei Sekundäranalysen typisch, lag der Fokus der Primärerhebung nicht spezifisch auf den Aspekten des Lebensendes, weshalb auch die Interviewführung nicht entsprechend vertiefende Nachfragen evozierte. Dafür konnte mit den Daten der Primärerhebung eben auch Perspektiven einbezogen werden von Personen, die sich mit dem Thema Vorsorge für das Lebensende noch wenig bzw. nicht auseinandergesetzt hatten. Einbußen in der Datenqualität konnten durch die Nacherhebung etwas ausgeglichen werden [20]. Die Erhebungen fanden zeitlich vor Eintreten der COVID-19-Pandemie statt. Schließlich gilt es bei der Einordnung der Ergebnisse zu beachten, dass die ÖIHS-Stichprobe einen überdurchschnittlich hohen Anteil an Personen mit akademischem Bildungsabschluss aufweist [13].

## Ausblick

Die Ergebnisse dieser Studie verdeutlichen die Dringlichkeit, Vorsorgemaßnahmen für alleinlebende hochaltrige Menschen zu verbessern, damit ihre Anliegen in Krisensituationen und am Lebensende berücksichtigt werden können. Die identifizierten Barrieren verweisen auf einen Forschungs- und Entwicklungsbedarf: Es geht darum, die vielschichtigen Anliegen der Betroffenen zur Sprache zu bringen und so aufzunehmen, dass dies auch als Richtschnur in Notfall- und Krisensituationen dienen kann. Ebenso ist noch ungelöst, wie solche Prozesse bzw. Angebote bestmöglich in der Primärversorgung etabliert werden können.

## Fazit für die Praxis

- Die Anliegen von alleinlebenden Menschen im häuslichen Umfeld gehen über gesundheitliche Vorausplanung hinaus und beziehen sich auf Aspekte von Pflegevorsorge sowie andere Aspekte rund um das Lebensende.
- Damit alleinlebende Menschen ihre Anliegen besprechen können, bedarf es regelhafter niederschwelliger Angebote der Vorsorge für das Lebensende.

## Data Availability

Daten aus Erhebungen der ÖIHS-Studie können im Rahmen einer Datennutzungsvereinbarung vom Österreichischen Institut für Alternsfragen bereitgestellt werden (Anfragen an office@oepia.at). Daten der Nacherhebung sind aus Datenschutzgründen nicht öffentlich zugänglich (die Einwilligungserklärung sah keine Erlaubnis zur Weitergabe der Daten vor).
